# *OcomCSP12*, a Chemosensory Protein Expressed Specifically by Ovary, Mediates Reproduction in *Ophraella communa* (Coleoptera: Chrysomelidae)

**DOI:** 10.3389/fphys.2019.01290

**Published:** 2019-10-15

**Authors:** Chao Ma, Shaowei Cui, Zhenya Tian, Yan Zhang, Guangmei Chen, Xuyuan Gao, Zhenqi Tian, Hongsong Chen, Jianying Guo, Zhongshi Zhou

**Affiliations:** ^1^State Key Laboratory for Biology of Plant Diseases and Insect Pests, Institute of Plant Protection, Chinese Academy of Agricultural Sciences, Beijing, China; ^2^School of Plant Protection, Jilin Agricultural University, Changchun, China; ^3^Guangxi Key Laboratory of Biology for Crop Diseases and Insect Pests, Institute of Plant Protection, Guangxi Academy of Agricultural Sciences, Nanning, China

**Keywords:** chemosensory proteins, *Ophraella communa*, RNA interference, expression profiles, reproduction

## Abstract

Chemosensory proteins (CSPs) are considered to be the transporter linking odorant chemicals and receptors on sensory neurons. However, the extensive expression patterns of CSPs in insects suggest that CSPs are also involved in other physiological processes; the range of their functions, however, remains uncertain. In this study, we successfully characterized and cloned the CSP12 of *Ophraella communa* (*OcomCSP12*). The open reading frame of *OcomCSP12* encodes 131 amino acids, with four conserved cysteine residues. The expression patterns of *OcomCSP12* validated by quantitative real-time PCR (qRT-PCR) showed that *OcomCSP12* is specifically expressed in female ovary. Furthermore, compared with the control treatment, silencing *OcomCSP12* resulted in significantly reduced oviposition in females. Surprisingly, the knock-down rate of *OcomCSP12* exceeded 95% and remained depressed for more than 15 days, indicating that RNA interference (RNAi) was a suitable method for exploring the function of CSP12 in *O. communa.* These findings increase our understanding of the expression profile and function of the CSP gene family in insects.

## Introduction

Through evolution, insects have evolved sensitive and complex olfactory systems to discriminate among a large variety of chemicals in the external environment. The olfactory system serves vital roles in several insect life activities such as host-searching, laying eggs, and mating. The insect olfactory system is primarily composed of an antennal lobe in the brain and diverse distinct sensilla (Vosshall et al., 2000). Odorants enter the sensilla through the pores and diffuse through the sensillar lymph to the receptors, binding and activating these proteins to effect semiochemical transduction. A variety of accessory proteins in the sensilla lymph are involved in the transport process, including the odorant-binding proteins (OBPs) and the chemosensory proteins (CSPs). Previously, insect CSPs were also known as olfactory-specific protein D (OS-D) (McKenna et al., 1994) or sensory appendage proteins (SAPs) (Robertson et al., 1999). Since the first CSP was discovered in *Drosophila* (McKenna et al., 1994), many other CSPs have been identified in different insects using sequencing technology, revealing, for example, the presence of eight CSPs in *Anopheles gambiae* (Sánchez-Gracia et al., 2009), ten in *Mamestra brassicae* (Campanacci et al., 2001), 16 in *Bombyx mori* (Gong et al., 2007), and 20 in *Tribolium castaneum* (Richards et al., 2008).

Both OBPs and CSPs are small, highly soluble proteins, able to bind small molecules such as odorants and pheromones (Gong et al., 2012). In addition, both groups have low isoelectric points and high concentrations surrounding chemosensory neurons (Pelosi et al., 2006). However, CSPs share no sequence similarities with OBPs, and there are many differences between them, as follows. (1) CSPs (10–15 kDa) are usually smaller than OBPs (15–20 kDa) and (2) there are four conserved cysteines in CSPs, while classic OBPs usually contain six conserved cysteines. Also, the four conserved cysteines found in CSPs are connected by two pairs of non-interlocked disulfide bridges (Wanner et al., 2004), while the six conserved cysteines in OBPs are connected by three interlocked disulfide bridges (Leal et al., 1999; Sandler et al., 2000). (3) Compared with OBPs, CSPs are more conserved in diverse insect species (Gu et al., 2012). (4) OBPs are mainly expressed in antennae, but CSPs can be found in most chemosensory organs, including antennae and maxillary palps (Maleszka and Stange, 1997), labial palps (Jin et al., 2005), and the proboscis (Meillour et al., 2000).

Compared with OBPs, CSPs are a poorly understood protein family. Because the protein properties of CSPs and OBPs are similar and both have high concentrations in sensillum lymph, it has been suggested that such soluble proteins may serve an important role in transporting odorants to their receptors on dendrite membranes (Picimbon et al., 2001; Pelletier and Leal, 2011). However, previous studies have reported that CSPs are also broadly expressed in non-chemosensory tissues (Lu et al., 2007), and the function of most CSPs is unknown. For example, p10 was isolated from regenerating legs in *Periplaneta americana*, where it was associated with the epidermis (Kitabayashi et al., 1998), p. 14 was isolated from the subcuticular layer in *Eurycantha calcarata* (Marchese et al., 2000), and several CSPs were purified from wings in *Locusta migratoria* (Ban et al., 2003). CSPs are also expressed in particular development stages, such as eggs, embryo larvae, pupa, and adults (Wanner et al., 2005; Foret et al., 2006; Qiao et al., 2013). The broad expression pattern of CSPs suggests that CSPs are related to diverse physiological functions beyond chemoreception in insects. For instance, CSPs are involved in embryonic development in honeybees (*Apis mellifera*) and diamondback moths (*Plutella xylostella*) (Maleszka et al., 2007; Gong et al., 2010). Gong et al. (2012) reported that silencing CSP3 has a negative effect on female survival and reproduction in *Spodoptera exigua*. Thus, it appears that CSPs play various non-olfactory roles, and the exact functions of CSPs need to be elucidated.

*Ophraella communa* Lesage (Coleoptera: Chrysomelidae) is considered to be an effective biological control agent of common ragweed, *Ambrosia artemissiifolia* L (Zhou et al., 2014). *O. communa* is present in the United States, Canada, Mexico, Japan, South Korea, Italy, France, China, and, in some locations, is believed to significantly suppress the *A. artemissiifolia* population (Chen et al., 2018). It is therefore important to explore the olfactory system and reproduction regulation mechanism of *O. communa.* To date, however, the functions of CSPs in this beetle have not been examined. In the present study, we first identified and cloned the ovary-specific CSP12 gene in *O. communa* (*OcomCSP12*). In addition, we silenced *OcomCSP12* in female beetles using RNAi technology, and the number of eggs laid was determined. Our findings on the function of CSP12 in *O. communa* broaden our understanding of the function of the CSP gene family in insects.

## Materials and Methods

### Insect-Rearing and Collection of Sample Tissues

*Ophraella communa* insects were obtained from the Institute of Plant Protection, China Academy of Agriculture Sciences. Beetles were reared on *A. artemissiifolia* plants at 26 ± 1°C, 75% RH, and a 14:10 h (light:dark) photoperiod in the laboratory. The ovaries were collected from five virgin female adults. In addition, to study gene expression profiles in different tissues, samples were collected of male antennae (M-A), female antennae (F-A), female ovaries (OV), male testis (TE), female heads (F-H), male heads (M-H), female legs (F-L), male legs (M-L), female thoraces (F-T), male thoraces (M-T), female wings (F-W), and male wings (M-W) from virgin adults of both sexes within 3 days of adult eclosion. All samples were immediately frozen in liquid nitrogen and stored at −80°C until RNA extraction.

### RNA Extraction, cDNA Synthesis, and Cloning of *OcomCSP12*

Total RNA was extracted from the five female ovaries collected, using TRIzol Reagent (Invitrogen, United States) and following the manufacturer’s protocols. The first strand of complementary DNA (cDNA) was synthesized from 1 μg total RNA using a first-strand cDNA Synthesis Kit (Transgen Biotech, Beijing, China) according to the manufacturer’s protocols. The synthesized cDNAs were stored at −20°C until use. To identify the OcomCSP12 sequence, a pair of degenerate primers (in Table 1) were designed to amplify the nucleic acid sequence. The polymerase chain reaction (PCR) was performed under the following thermal program: 94°C 5 min, 35 cycles of 94°C for 30 s, 48°C for 30 s, and 72°C for 90 s, followed by one cycle at 72°C for 10 min. The PCR product was purified with a Monarch gel extraction kit (NEB, England) and cloned into a Trans1-T1 clone vector (Transgen Biotech, Beijing, China) and sequenced (Sangon Biotech, Shanghai, China).

**TABLE 1:** List of design primers for PCR for *OcomCSP12* cloning, qRT-PCR, and dsOcomCSP12 synthesis.

**Primer name**	**Sequence (5′–3′)**
*OcomCSP12*-F	ATGAAATTCGCTGTTTTT
*OcomCSP12*-R	TTAAAATTGTATTCCAGC
*OcomCSP12*-F-RT	CTACGTCGATTGTCTGGT
*OcomCSP12*-R-RT	CGCTTCTTTTTTCTGTTT
*RL19*-F	AAGGAAGGCATTGTGGAT
*RL19*-R	GACGCAAATCTCGCATAC
dsOcomCSP12-F	TAATACGACTCACTATAGGGAATTCGCTGTTTTTCTTT
dsOcomCSP12-R	TAATACGACTCACTATAGGGTTTCATTCCACCAGTCTC
dsEGFP-F	TAATACGACTCACTATAGGGTGAGCAAGGGCGAGGAG
dsEGFP-R	TAATACGACTCACTATAGGGCGGCGGTCACGAACTCCAG

### Sequence Analysis and Phylogenetic Tree Construction

Amino acid sequences of *OcomCSP12* were translated using ExPASy^1^, and the CSPs of other insect species discussed in this study were retrieved from the NCBI database^2^. Molecular weight, isoelectric point, signal peptide, and the occurrence of α-helices were predicted through ExPASy^3^. Multiple sequence alignment was carried out with DNAMAN software (LynnonBiosoft, United States), and the comparison graph was generated with WebLogo^4^. The phylogenetic tree was constructed with MEGA 7.0 software^5^ using the neighbor-joining method, and the tree was reconstructed with 1000-replicate bootstrap analysis.

### Expression Profiles of *OcomCSP12*

The expression profiles of *OcomCSP12* were analyzed using qRT-PCR. Total RNA was isolated from the twelve different tissues as described above. The concentration of each RNA sample was standardized to 1 μg/ul, and the cDNA was synthesized using a first-strand cDNA synthesis kit for qPCR (Transgen Biotech, Beijing, China) according to the manufacturer’s protocol. *Ribosomal protein* (*RL19*) was used as an internal control. The qRT-PCR was performed using ABI 7500 (Thermo Scientific, Waltham, MA, United States) with TransStar Tip Top Green qPCR Supermix (Transgen Biotech, Beijing, China). The PCR reaction programs were 30 s at 94°C, 40 cycles of 94°C for 5 s, and 60°C for 34 s. The qRT-PCR primers were designed using Primer Premier 5.0 (PREMIER Biosoft International), and the efficiency of primers was validated before gene expression analysis. All qRT-PCR primer sequences are listed in Table 1. Each qRT-PCR reaction was performed using three technical replicates and three biological replicates.

### DsRNA Synthesis, Microinjection, and qRT-PCR Validation

Primers used to amplify the double-strand RNA (dsRNA) of the OcomCSP12 gene (dsOcomCSP12) and the enhanced green fluorescent protein (dsEGFP) (as the control treatment) are listed in Table 1. All the primers were designed with Primer Premier 5.0 (PREMIER Biosoft International), and the length of dsOcomCSP12 fragment was 352 bp. After PCR amplification, the targeted fragment obtained was used to synthesize dsRNA with a MEGAscript RNAi kit (Ambion Inc., United States) according to the manufacturer’s instructions. The concentrations of the synthesized dsOcomCSP12 and dsEGFP were diluted to 10 μg/ul and stored at −20°C until use.

Newly emerged females of *O. communa* (<12 h) were used for RNA interference (RNAi) microinjection to explore the function of *OcomCSP12* relative to female reproduction. To limit insect movement, an agarose plate placed on an ice tray was used to immobilize the insects. Then, 0.1 μl aliquots each of dsOcomCSP12 and dsEGFP were injected into the abdomen of each *O. communa* female, using a PLI-100 Pico-Injector (Harvard Apparatus, Holliston, MA, United States) manipulated by an MP-255 Micromanipulator (Sutter, Novato, CA, United States) under a microscope. After injection, injected females were paired with untreated males of the same age as the females. Each petri dish contained only one pair of beetles, where were fed with fresh leaves of *A. artemissiifolia*. Forty individuals were used for oviposition assay, and this assay was repeated three times.

To better investigate the function of *OcomCSP12*, we collected the ovaries of injected females 2, 5, 10, and 15 days after injection to validate the efficiency of gene silencing. The *OcomCSP12* mRNA levels were determined by qRT-PCR, and all procedures were performed as described above.

### Oviposition Assay

After injection, the number of eggs laid per female was counted every day for 15 consecutive days, and fresh leaves of *A. artemissifolia* were provided every day. During this period, if the male died, an untreated male that had emerged at the same time as the female was provided as a replacement, and we continued to record the number of eggs laid. Only females that survived for all 15 days were used for analysis.

### Data Analysis

qRT-PCR and oviposition data were analyzed with SAS 9.0 (SAS Institute Inc., Cary, NC, United States). qRT-PCR data were analyzed using the 2^–ΔΔ*CT*^ method. Differences among treatments were evaluated by analysis of variance (ANOVA) using an LSD test at a significance level of *P* < 0.05. Figures were made using OriginPro 9.1 (Northampton, Massachusetts, United States).

## Results

### Identification and Sequence Analysis of *OcomCSP12*

**FIGURE 1:**
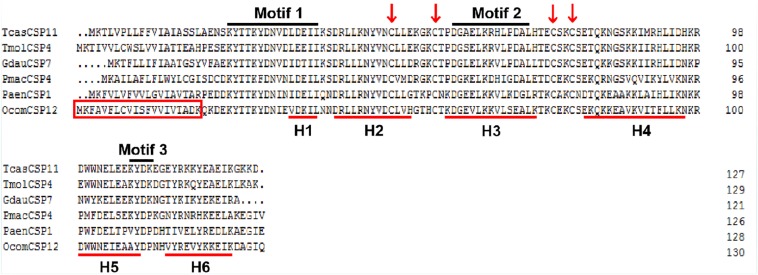
Alignment of the complete amino acid sequence of *OcomCSP12* and five other CSPs of coleopteran insects. The signal peptides of *OcomCSP12* are in the red box; the four conserved cysteine residues are marked with a red arrow; the six predicted α-helices are indicated by red underlines; three conserved motifs are under black lines. Tcas, *Tribolium castaneum*; Tmol, *Tenebrio molitor*; Gdau, *Galeruca daurica*; Pmac, *Pyrrhalta maculicollis*; Paen, *Pyrrhalta aenescens*.

The full-length *OcomCSP12* gene that we cloned contained a 396 bp open reading frame, encoding a polypeptide of 131 amino acids (GenBank number: MN296017). The molecular weight of *OcomCSP12* was 15.39 kDa, and the isoelectric point was 8.71. *OcomCSP12* contained a signal peptide of 20 residues at the N-terminal and six α-helices (H1-H6) (Figure 1). There were four conserved cysteine residues in *OcomCSP12*, which agrees with the classical model of Cys-X_6__–__8_-Cys-X_16__–__21_-Cys-X_2__–__4_-Cys (Figure 1). According to Wanner et al. (2004) and Li et al. (2011), *OcomCSP12* also has three highly conserved sequence motifs, including Motif 1: N-terminal YTTKYDN(V/I)(N/D)(L/V)DEIL, Motif 2: central DGKELKXX(I/L)PDAL, and Motif 3: C-terminal KYDP (Figure 1).

**FIGURE 2:**
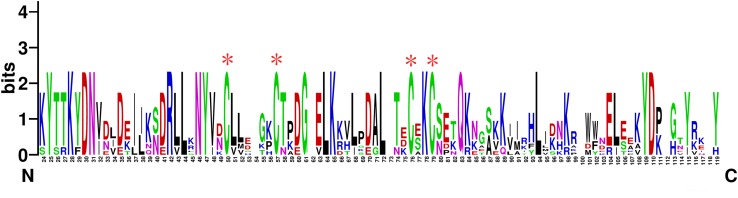
The WebLogo alignment of *OcomCSP12* and five other CSPs of coleopteran insects. ^∗^Four highly homologous cysteines; N, N-terminal, C, C-terminal.

**FIGURE 3:**
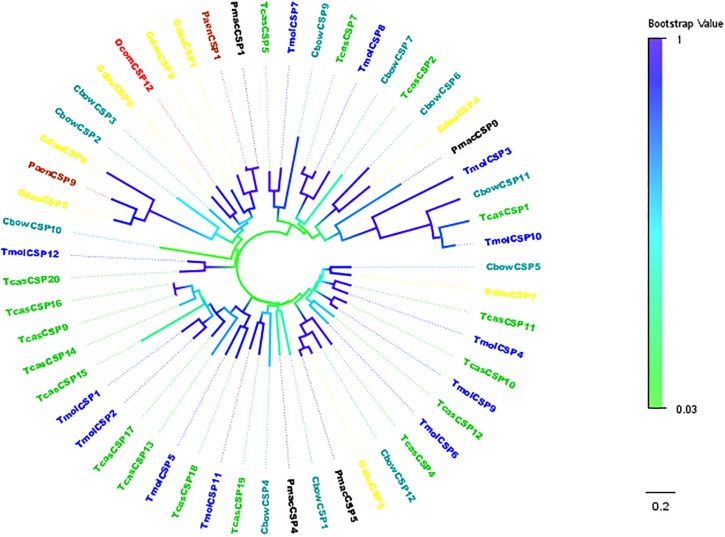
Neighbor-joining phylogenetic tree of *OcomCSP12* (in red) with known Coleopteran CSP sequences. Tcas, *Tribolium castaneum* (in green); Pmac, *Pyrrhalta maculicollis* (in black); Paen, *Pyrrhalta aenescens* (in wine); Gdua, *Galeruca daurica* (in yellow); Cbow, *Colaphellus bowringi* (in dark cyan); Tmol, *Tenebrio molitor* (in blue).

*TcasCSP11*, *Tmol CSP4*, *GdauCSP7*, *PmacCSP4*, and *PaenCSP1* were chosen for multiple sequence alignment with *OcomCSP12* and *OcomCSP12*, which displayed about 50% similarity to these other genes. The WebLogo graph generated for the six CSPs showed that there were four completely conserved cysteines at positions 50, 57, 76, and 79; other positions such as 25 (Y), 28 (K), 30 (D), 31 (N), 42 (R), 44 (L), 46 (N), 47 (Y), 61 (G), 64 (L), 65 (K), 72 (L), 83 (Q), 94 (L), 109 (Y), and 110 (D) are also highly conserved (Figure 2), indicating that CSP is a class of highly conserved protein. The results of the phylogenetic tree analysis showed that, for coleoptera insects, CSP genes consist of three branches, and *OcomCSP12*, *GdauCSP6*, *GdauCSP8*, *GdauCSP1*, *PaenCSP1*, and *PmacCSP1* cluster into one group, supported by high bootstrap values (Figure 3).

### Tissue Distribution of *OcomCSP12*

**FIGURE 4:**
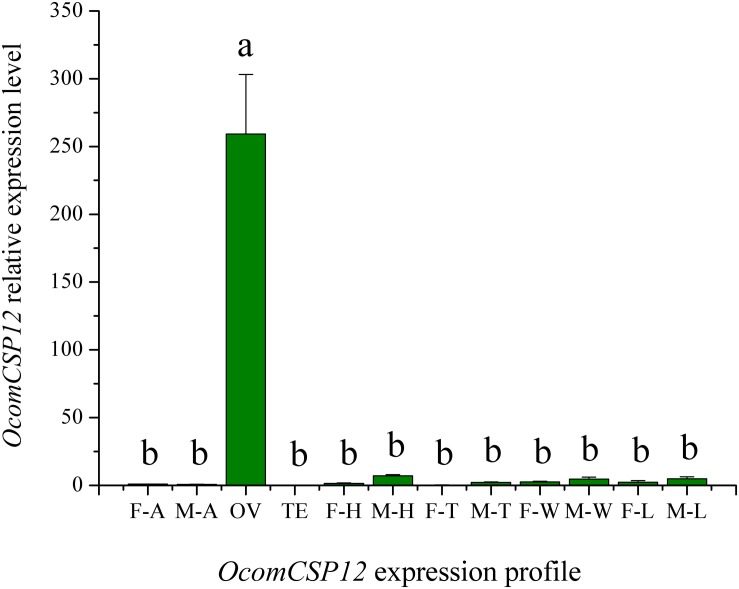
Expression pattern of *OcomCSP12* in different male and female tissues. F-A, female antennae; M-A, male antennae; OV, female ovaries; TE, male testis; F-H, female heads; M-H, male heads; F-T, female thoraces; M-T, male thoraces; F-W, female wings; M-W, male wings; F-L, female legs; M-L, male legs. All expression fold changes are related to female antennae. Bars with the same letter are not significantly different from each other at *P* < 0.05 based on LSD test. Each point represents mean ± SE.

The expression level of *OcomCSP12* transcripts in male antennae (M-T), female antennae (F-T), female ovaries (OV), male testis (TE), female heads (F-H), male heads (M-H), female legs (F-L), male legs (M-L), female thoraces (F-T), male thoraces (M-T), female wings (F-W), and male wings (M-W) were measured by qRT-PCR, and *RL19* was used as the internal control. The qRT-PCR results showed that *OcomCSP12* had extremely low expression in male and female antennae and that there was no significant difference between the sexes (Figure 4). Among the different issues of male and female beetles, *OcomCSP12* expressed in ovary was significantly higher than that expressed in other tissues (*F* = 34.03, *P* < 0.0001), and there were no significant differences among expression levels in other tissues, suggesting that *OcomCSP12* was specifically expressed in the female ovary (Figure 4).

### Silence-Efficiency Validation and the Effect of *OcomCSP12* on Female Reproduction

**FIGURE 5:**
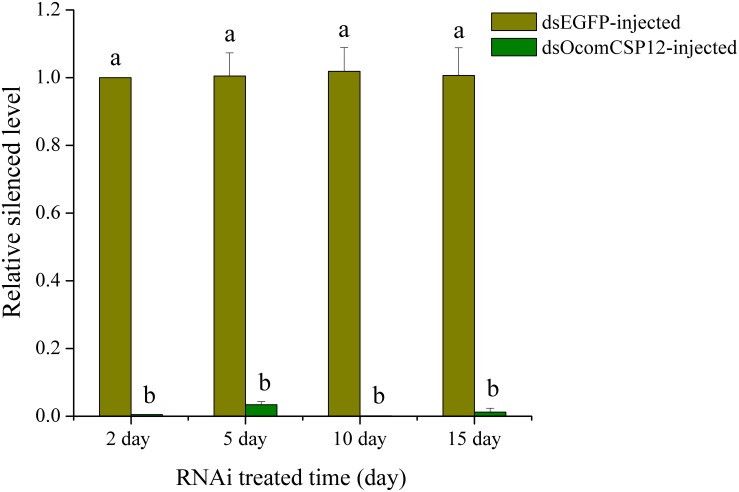
Validation of *OcomCSP12*-silencing level in dsOcomCSP12-injected and dsEGFP-injected over time following RNAi treatment. The expression level of *OcomCSP12* was normalized to the *RL19* control gene. Values marked with different letters are significantly different based on one-way analysis of variance (ANOVA) and LSD test.

**FIGURE 6:**
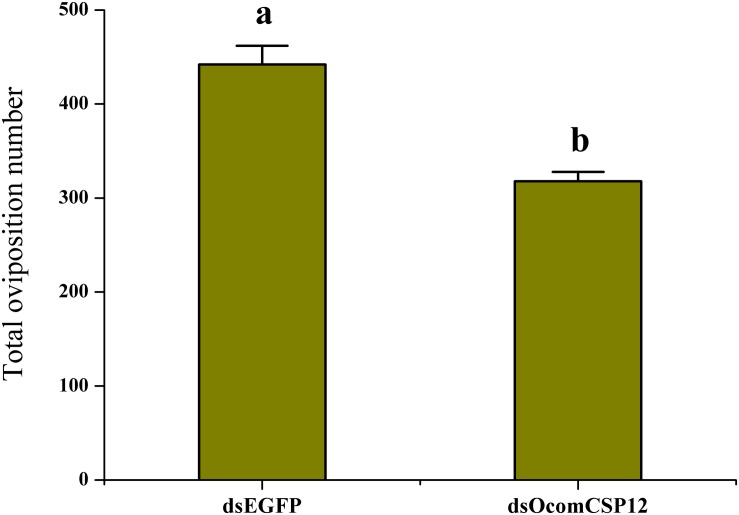
Effect of *OcomCSP12* silencing on the reproduction of female *Ophraella communa*. Values marked with different letters are significantly different based on one-way analysis of variance (ANOVA) and LSD test.

As part of our study of the role of *OcomCSP12* in female reproduction, we silenced this gene using RNAi. We validated the efficacy and duration of the silencing effect 2, 5, 10, and 15 days after injection. Compared with insects injected with dsEGFP (as a control treatment), in insects injected with the silencing element dsOcomCSP12, the level of *OcomCSP12* transcripts remained extremely low at all four post-injection time points, with a knock-down rate of >95% (Figure 5). This means that an interference effect could exist in *O. communa* for more than 15 days with high silencing efficiency, suggesting that RNAi technology is suitable for studying the function of *OcomCSP12* in *O. communa*. The total number of eggs laid by RNAi-treated females 15 days after injection was significantly lower than those laid by control females, being reduced by about 28% (*F* = 35.33, *P* < 0.0001; Figure 6).

## Discussion

At present, CSPs are known to be widely distributed in more than ten orders of insects (Gong et al., 2010). A CSP-like protein was identified in the brine shrimp *Artemia franciscana*, indicating that CSPs may exist across the arthropods (Pelosi et al., 2006). CSPs are a family of ancient and highly conserved proteins. Foret et al. (2006) reported that the CSP family is not only relatively well conserved in various insect species but also appears to have even more ancient roots in the arthropods. CSPs have four conserved cysteines linked by disulfide bridges between neighboring residues and show a novel type of α-helical structure, with six helices connected by α-α loops (Wanner et al., 2004). Our data (Figure 1) demonstrate that *OcomCSP12* has these sequence and structural characteristics, indicating that *OcomCSP12* belongs to the CSP family.

Due to CSPs having binding activity and high abundance around the dendrites of olfactory neurons, they have been supposed to bind odorant molecules and transfer them to odorant receptors, as OBPs do. Some studies have reported binding of insect CSPs with host plant volatiles, sex pheromone analogs, and other related compounds (Dani et al., 2011; Gu et al., 2012; Iovinella et al., 2013; Sun et al., 2014; Yi et al., 2014; Zhang et al., 2014). Recently, Lin et al. (2018) found that CSPs in *Spodoptera litura* can direct binding pesticides to adapt to the environment. In contrast to OBPs, which are mainly expressed in olfactory-related tissues, CSPs have a broad expression pattern, including expression in the antennae, head, thorax, legs, wings, testes, ovaries, epithelium, and pheromone glands (Gong et al., 2007; Lu et al., 2007), suggesting that CSPs may participate in various processes. In the present study, however, qRT-PCR data (Figure 4) showed that *OcomCSP12* is specifically expressed in female ovary and is present at only an extremely low rate of transcript in other tissues of females and males, suggesting that *OcomCSP12* may play a role in reproduction. Subsequent treatment with RNAi (Figure 6) confirmed that compared with the control treatment, silencing *OcomCSP12* significantly reduced the oviposition of female beetles. Similarly, Gong et al. (2012) reported that silencing CSP3 in *S. exigua* sharply reduced female survival and reproduction. That CSPs are related to embryo development has been confirmed (Maleszka et al., 2007). In general, CSPs are involved in insect reproduction when the CSPs are specifically expressed in the reproductive tissues, such as is the case for *BmorCSP12* and *BmorCSP7*, which are expressed in the ovaries of *Bombyx mori* (Gong et al., 2007). In addition, Maleszka et al. (2007) found that CSP5 in *Apis mellifera* was only expressed in ovary and eggs and is directly involved in embryonic integument formation. This means that interfering with the transcript level of *OcomCSP12* may affect oogenesis or embryogenesis, thus significantly reducing the number of eggs laid by treated female beetles.

However, the extensive expression pattern of CSPs makes it likely that they are also involved in other, diverse physiological processes that are related to neither reproduction nor olfaction. In *Periplaneta americana*, the content of p10 in a regenerating leg was significantly higher than that in normal legs, suggesting that p10 may promote regeneration (Kitabayashi et al., 1998). In addition, when flies were infected by viruses or bacteria, the expression levels of pebIII and phk3 in *Drosophila melanogaster* were significantly increased (Sabatier et al., 2003). Clearly, further research is required to determine the specific functions of CSPs at the molecular level, and this study will provide clues to better understand the function of CSPs in insects.

## Data Availability Statement

The datasets generated for this study can be found in the NCBI using accession MN296017.

## Ethics Statement

The animal study was reviewed and approved by Ethics of Animal Experiments of the IPP, CAAS.

## Author Contributions

CM and ZZ designed the research, conducted the experiments, analyzed the data, and wrote the manuscript. SC, ZYT, YZ, GC, XG, and ZQT participated in the sample collection and data recording. HC, JG, and ZZ revised the manuscript. All authors contributed to the research design, manuscript preparation, read, and approved the final manuscript.

## Conflict of Interest

The authors declare that the research was conducted in the absence of any commercial or financial relationships that could be construed as a potential conflict of interest.

## Footnotes

1 https://web.expasy.org/translate/

2 https://www.ncbi.nlm.nih.gov/

3 http://www.expasy.org/

4 http://weblogo.berkeley.edu/logo.cgi/

5 http://www.megasoftware.net/
